# Aminoglycoside Drugs as Adjuvants to Enhance siRNA/mRNA Delivery by Lipid Nanoparticles

**DOI:** 10.1021/acsomega.5c05346

**Published:** 2025-11-03

**Authors:** Xueru Sun, Lei Qian

**Affiliations:** † Wulanchabu Medical College, Wulanchabu, Inner Mongolia 012000, China; ‡ Affiliated Hospital of Wulanchabu Medical College, Wulanchabu, Inner Mongolia 012000, China

## Abstract

Nucleic acid drugs (such as siRNA and mRNA) hold broad prospects in disease treatment, but their clinical application is limited by low delivery efficiency, particularly due to insufficient lysosomal escape. This study aims to explore aminoglycoside small-molecule drugs as adjuvants to enhance the lysosomal escape efficiency of lipid nanoparticles (LNPs) for nucleic acid drug delivery, thereby improving therapeutic effects and providing new strategies for gene therapy. In this study, five FDA-approved aminoglycoside small molecules were selected as adjuvants to enhance the lysosomal escape of LNPs/siRNA and improve the siRNA delivery effect. *In vitro* results confirmed that aminoglycoside drugs significantly enhanced the delivery efficiency of LNPs/siRNA, reducing luciferase expression to as low as 22.15%. Mechanistic studies revealed that kanamycin sulfate markedly reduced siRNA colocalization with lysosomes and destabilized lysosomal membranes under acidic conditions (pH 6.2), increasing hemolytic effects by 3.6-fold. Additionally, kanamycin sulfate significantly improved the silencing efficacy of LNPs/siEGFR on EGFR mRNA and protein expression and boosted LNPs/mSARS-CoV-2-mediated spike protein expression. Therefore, aminoglycoside small molecules (particularly kanamycin) can effectively enhance nucleic acid delivery efficiency by disrupting endosomal membrane structures, providing a novel adjuvant strategy for the clinical application of siRNA and mRNA drugs.

## Graphical abstract

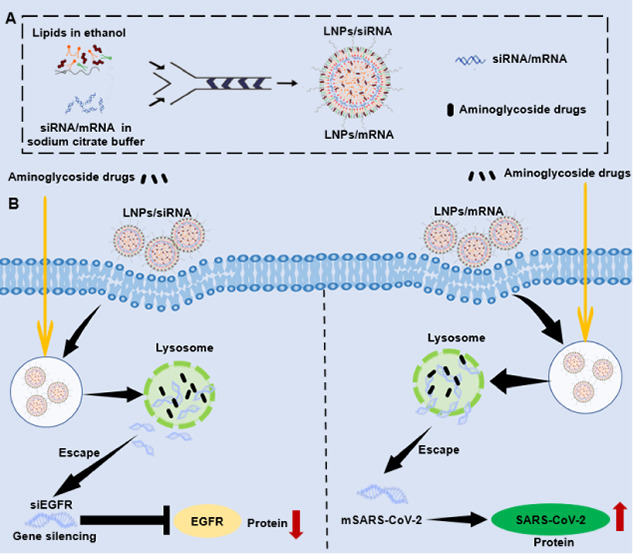

## 1 Introduction

Nucleic acid drugs (such as siRNA, mRNA, and antisense oligonucleotides) have emerged as cutting-edge tools for treating genetic diseases, cancers, and viral infections due to their potential to precisely regulate gene expression.^1^ For example, small interfering RNA (siRNA) and messenger RNA (mRNA) can both modulate intracellular protein expression.^2–4^ After cytoplasmic delivery, siRNA activates the RNA interference (RNAi) pathway, leading to sequence-specific gene silencing at the post-transcriptional level, while mRNA delivery can drive the expression of therapeutic proteins and antigens.^5–7^ However, their clinical application still faces the critical bottleneck of low delivery efficiency. With the approval of siRNA drugs like Onpattro and Givlaari, lipid nanoparticles (LNPs) have become one of the most promising carriers for the clinical translation of gene therapy.^8^ Additionally, the recent approval of Pfizer/BioNTech and Moderna’s LNPs based mRNA vaccines for SARS-CoV-2 highlights the immense potential of LNPs in mRNA vaccines for infectious disease prevention.^9,10^ Furthermore, the success of mRNA vaccines is expected to accelerate the therapeutic applications of mRNA. Nevertheless, the clinical use of LNPs based siRNA/mRNA delivery still faces challenges, particularly insufficient lysosomal escape. Although LNPs employ various lysosomal escape strategies (e.g., lysosomal membrane fusion or disruption) to promote siRNA endosomal escape, this process remains largely inefficient, with the majority of siRNA degraded in lysosomes. Typically, less than 1%–2% of siRNA is released into the cytosol, far below the requirements for clinical applications.^4,11^ Therefore, developing novel strategies to enhance the lysosomal escape efficiency of nucleic acid drugs is a core issue for improving their clinical translation.

In recent years, small-molecule drug assisted delivery technology has garnered significant attention due to its controllability and ease of modification. Some researchers have screened 56 cationic amphiphilic drugs (CADs) and found that certain molecules can transiently induce endosomal/lysosomal membrane permeabilization, promoting the release of functionally captured siRNA.^12^ Aminoglycoside compounds (e.g., kanamycin sulfate, gentamicin sulfate, and tobramycin sulfate) are clinically used to treat severe infections by inhibiting bacterial protein synthesis.^13^ Their structures consist of an aminocyclitol ring linked to one or more amino sugar molecules via glycosidic bonds.^14^ As a class of highly positively charged polyamine small molecules, they not only exhibit strong electrostatic interactions with negatively charged nucleic acids but can also accumulate in cellular lysosomes, potentially disrupting lysosomal membrane structures to enhance nucleic acid drug escape and improve delivery efficiency.^15^

Based on this, our study explores the use of aminoglycoside small molecules as adjuvants to enhance LNPs based nucleic acid drug lysosomal escape and delivery efficiency. We systematically evaluate the effects of various aminoglycoside small molecules on intracellular nucleic acid transport efficiency and further elucidate their mechanisms for promoting endosomal escape. At the clinical application level, we validate the enhanced delivery effects using antitumor EGFR siRNA and SARS-CoV-2 mRNA to demonstrate the broad-spectrum efficacy and clinical translational value of aminoglycoside adjuvants. In summary, this study aims to provide a novel adjuvant strategy for nucleic acid drug delivery systems.

## 2 Materials and Methods

### 2.1 Materials

Human nonsmall cell lung cancer cells (A549), luciferase-labeled human nonsmall cell lung cancer cells (A549-Luc), human embryonic kidney epithelial cells (HEK293T), and other cell lines were obtained from the Chinese Academy of Medical Sciences Tumor Cell Bank. Streptomycin sulfate, gentamicin sulfate, kanamycin sulfate, tobramycin sulfate, and amikacin sulfate were purchased from Shanghai Yuanye Biotechnology Co., Ltd. Ethanol and methanol were obtained from Beijing Tongguang Fine Chemical Company. Nuclease-free water was sourced from Baisha Biotechnology Co., Ltd. Dlin-MC3-DMA, DSPC, cholesterol (Chol), and DMG-PEG2000 were provided by AVT (Shanghai) Pharmaceutical Technology Co., Ltd. Negative control siRNA (F: 5′-UUCUCCGAACGUGUCACGUTT-3′, R: 5′-ACGUGACACGUUCGGAGAATT-3′), EGFR-siRNA (F:5′-AGGAAUUAAGAGAAGCAACAU-3, R: 5′-AUGUUGCUUCUCUUAAUUCC U-3′), FAM-siRNA, Cy5-siRNA, and siLuc (human; F: 5′-GAUUAUGUCCGGUUAUGUATT-3′; R: 5′-UACAUAACCGGACAUAAUCTT-3′) were synthesized by Suzhou Gene Pharma Co., Ltd. PBS buffer and dialysis bags (MW 20000) were purchased from Beijing Solarbio Science & Technology Co., Ltd. Dimethyl sulfoxide (DMSO, chromatographic grade) was obtained from Sigma-Aldrich. LysoTracker Red DNA-99 and Hoechst 33342 were sourced from Beijing Xinxiyuan Biotechnology Co., Ltd. Disposable sterile syringes (1 mL) were purchased from Shanghai BD Medical Devices Co., Ltd. SQP electronic analytical balance was obtained from Beijing Sartorius Co., Ltd. D-400898 syringe pump was purchased from Harvard Apparatus (USA). XW-80A vortex oscillator was obtained from Jiangsu Haimen Qilin Bell Instrument Manufacturing Co., Ltd. 20 mm confocal dishes were purchased from MatTek (USA). Cell culture flasks (25 cm^2^), 96-well plates, 6-well plates, 12-well plates, and pipettes were sourced from Corning (USA).

### 2.2 Preparation of LNP/siRNA or LNP/mRNA Lipid Nanoparticles

The organic phase was prepared by dissolving lipids (Dlin-MC3-DMA:Chol:DSPC:DMG-PEG = 50:38.5:10:1.5 molar ratio) in ethanol. The aqueous phase was prepared by diluting siRNA/mRNA in nuclease-free water. The organic and aqueous phases were mixed at a 1:3 flow rate (total flow rate = 4 mL/min) using a microfluidic device. The mixture was collected and dialyzed overnight in PBS using a dialysis bag (MW 20,000) to obtain LNPs/siRNA or LNPs/mRNA lipid nanoparticles (final concentration: 1 μM, N/P = 6).

### 2.3 CCK8 Assay for Aminoglycoside Drug Toxicity

A549-Luc cells were seeded in 96-well plates at a density of 8,000 cells per well (200 μL DMEM-F12K medium) and cultured for 24 h. After cell attachment, the medium was replaced with fresh medium containing various concentrations of aminoglycoside drugs (streptomycin sulfate, gentamicin sulfate, kanamycin sulfate, tobramycin sulfate, or amikacin sulfate). Control wells received medium only. After 24 h, the drug-containing medium was removed, and 10% CCK8 solution in medium was added to each well. After 2 h of incubation, the absorbance (OD) at 450 nm was measured using a microplate reader. Cell viability (%) was calculated as (OD of treated wells – OD of blank wells)/(OD of control wells – OD of blank wells) × 100%.

### 2.4 The Impact of Aminoglycoside Drugs on the Efficacy of LNPs

For siRNA efficacy assessment, A549-Luc cells were seeded in 96-well plates (8,000 cells/well, 200 μL DMEM-F12K medium) and cultured for 24 h. After attachment, cells were treated with various concentrations of free small-molecule drugs (streptomycin sulfate, gentamicin sulfate, kanamycin sulfate, tobramycin sulfate, or amikacin sulfate). After 4 h, the medium was replaced with fresh medium containing 25 nM LNPs/siLuc nanoparticles. After 24 h, the medium was removed, and cells were washed with PBS. Luciferase substrate (100 μL) was added, and after 15 min of shaking, luminescence was measured using a BioTek Synergy Neo2 microplate reader. Relative luciferase activity was calculated as (luminescence of treated group – blank luminescence)/(luminescence of control group–blank luminescence).

### 2.5 Kanamycin Sulfate on Lysosomal Escape Assay of siRNA

A549 cells were seeded in 20 mm glass-bottom dishes (200,000 cells/dish) and cultured for 24 h. After attachment, cells were treated with medium containing different concentrations free kanamycin sulfate for 4 h. The medium was then replaced with DMEM-F12K medium containing Cy5-siRNA-loaded LNPs/siRNA (final concentration: 50 nM). After 4 or 8 h of incubation, cells were stained with LysoTracker Red (300 nM) for 30 min, fixed with 4% paraformaldehyde, and stained with Hoechst 33342 (10 μg/mL). Cells were imaged using a Zeiss LSM880 confocal microscope (Hoechst 33342: Ex/Em = 346/460 nm; Cy5: Ex/Em = 650/670 nm; LysoTracker Red: Ex/Em = 567/590 nm.

### 2.6 Kanamycin Sulfate on Lysosomal Membrane Rupture Assay

Red blood cells (RBCs) were isolated from fresh citrate-treated mouse blood, washed first in phosphate-buffered saline (PBS), and then in lysis assay buffer (20 mM HEPES-HCl, 150 mM NaCl, pH 7.4 or 6.2). The RBC pellet was resuspended in 10 volumes of lysis assay buffer. In a 96-well tissue culture plate, either kanamycin sulfate-free or kanamycin sulfate (1 mM) and LNPs/siRNA mixtures were added to 135 μL of lysis assay buffer, followed by mixing with 15 μL of RBC suspension. The plate was then incubated at 37 °C with continuous shaking for 1 h. Hemoglobin release was measured using a microplate UV–vis spectrophotometer (absorbance at 450 nm, Abs450). Complete RBC lysis was determined by treatment with 10% Triton X-100. The Abs450 of the lysis assay buffer alone was set as the negative control.

### 2.7 PCR Detection of EGFR Gene Silencing Effect

A549 cells were seeded in 12-well plates and cultured for 24 h to allow adherence. The medium was removed and replaced with OPTI-DMEM containing 25 nM siRNA in LNPs/siEGFR, LNPs/siNC, or LNPs/siEGFR combined with kanamycin sulfate. The control group received medium only. After 6 h of transfection, the medium was replaced with complete DMEM. After 24 h, the medium containing the formulations was discarded, and EGFR mRNA expression was analyzed via RT-PCR.

### 2.8 ELISA Kit Detection of EGFR Protein Expression

A549 cells were seeded in 6-well plates and cultured for 24 h. The medium was replaced with OPTI-DMEM containing 25 nM siRNA in LNPs/siEGFR, LNPs/siNC, or LNPs/siEGFR combined with kanamycin sulfate, while the control group received medium only. After 6 h of transfection, the medium was replaced with complete DMEM medium. After 24 h, the medium was discarded, and EGFR protein levels were measured using an EGFR ELISA kit.

### 2.9 ELISA Kit Detection of S Protein Expression

HEK293T cells were seeded in 24-well plates at a density of 5 × 10^5^ cells/well and incubated overnight for 12 h. The medium was then replaced with LNPs/mSARS-CoV-2 containing 2 μg/mL mRNA, LNPs/mSARS-CoV-2 + Kanamycin mixture (2 μg/mL mRNA equivalent), Control wells received PBS-containing medium. After 6 h of transfection, all wells were replaced with fresh complete DMEM medium and cultured for 48 h. Cell supernatants were collected following centrifugation at 1000 g. SARS-CoV-2 S protein expression in HEK293T supernatants was quantitatively measured using a SARS-CoV-2 Omicron variant Spike ELISA detection kit.

### 2.10 Statistical Analysis

All data are presented as mean ± SD (x̅ ± s) and analyzed using GraphPad Prism 8.0. Comparisons among multiple groups were conducted using one-way ANOVA, while intergroup comparisons between two sets utilized *t* test. P* < 0.05 was considered statistically significant.

## 3 Results

### 3.1 Selection of Aminoglycoside Small-Molecule Drugs

We selected five FDA-approved pharmacologically active aminoglycoside small-molecule drugs: streptomycin sulfate (STR), gentamicin sulfate (GM), kanamycin sulfate (KAN), tobramycin sulfate (NN), and amikacin sulfate (AN). The chemical structure is shown in Figure 1.

**Figure 1:**
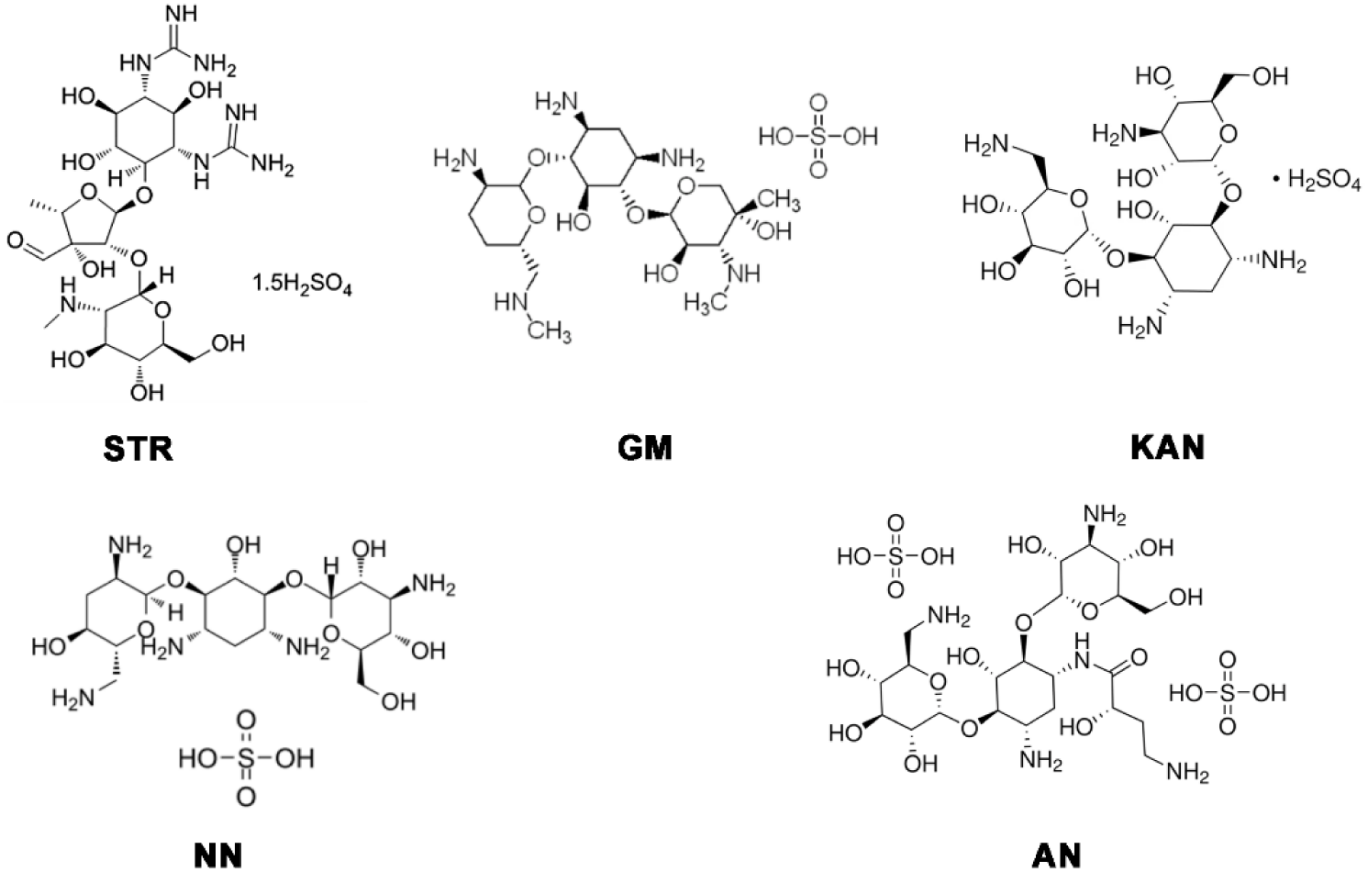
Molecular structures of aminoglycoside drugs.

### 3.2 Characterization of LNPs/siRNA Lipid Nanoparticles

Dynamic light scattering (Malvern Zetasizer) showed that the prepared LNPs/siRNA had an average particle size of 90.4 ± 0.52 nm, a zeta potential of −3.82 ± 0.00 mV, and a polydispersity index (PDI) of 0.245 (Figure 2A-B). TEM revealed uniformly spherical or near-spherical nanoparticles with a diameter of ∼90 nm (Figure 2C). These results confirm that LNPs/siRNA exhibit small size, negative surface charge, and low PDI.

**Figure 2:**
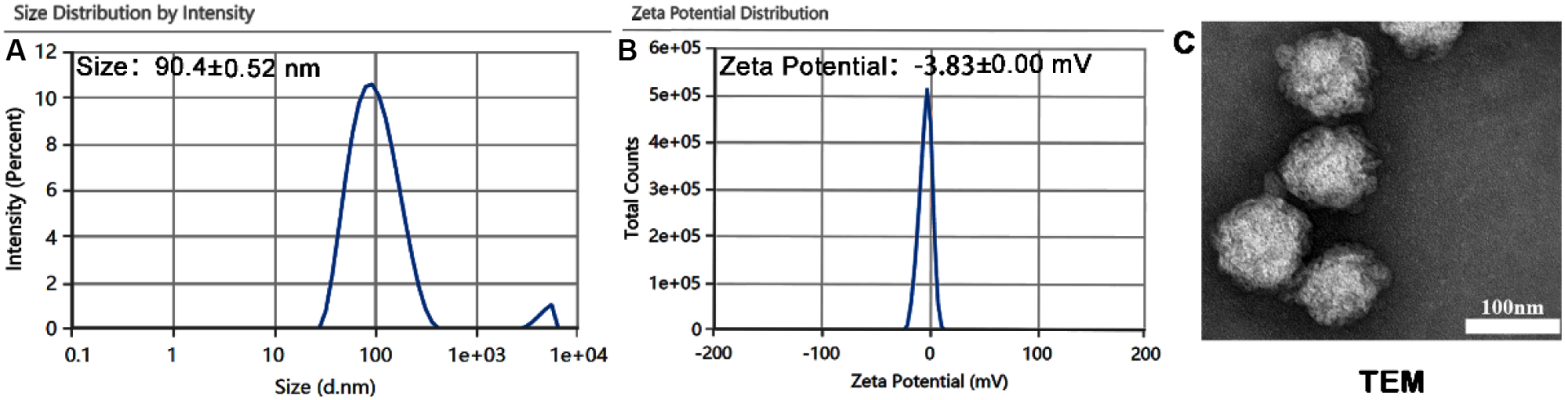
Characterization of LNPs/siRNA lipid nanoparticles: (A) DLS (volume distribution). (B) Zeta potential. (C) Transmission electron microscopy (TEM) images of LNPs/siRNA were shown. The scale bars represented 100 nm, and the magnification was set at 40,000×. The data presented as mean ± standard deviation (*n* = 3).

### 3.3 Toxicity of Aminoglycoside Drugs

Screening drugs at safe concentrations is essential for enhancing siRNA delivery. As shown in Figure 3, the nontoxic concentration ranges (cell viability >80%) were: STR: 20–2500 μM, GM: 20–2500 μM, KAN: 20–2500 μM, NN: 20–5000 μM, and AN: 20–2500 μM. These ranges were used for subsequent siRNA delivery enhancement assays.

**Figure 3:**
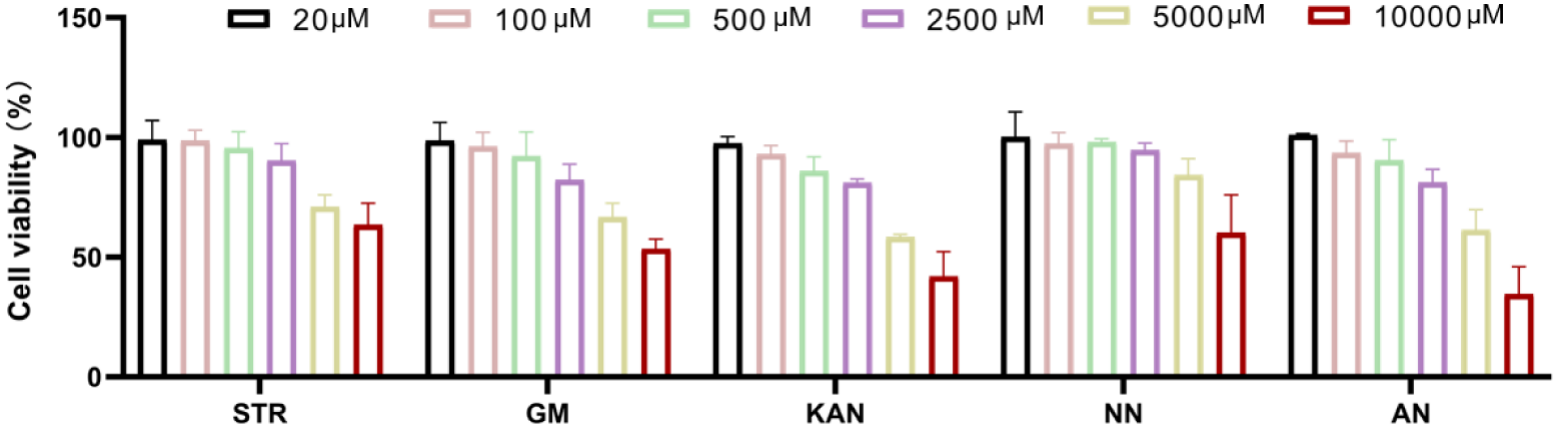
Toxicity of small molecule drugs at different concentrations. The data presented as mean ± standard deviation (*n* = 3).

### 3.4 Aminoglycoside Drugs Enhance LNPs/siLuc Gene Silencing

siRNA silences genes by targeting mRNA. LNPs/siLuc downregulates Luc-mRNA in A549-Luc cells, reducing luciferase expression for rapid RNAi screening. As shown in Figure 4, all tested aminoglycosides enhanced LNPs/siLuc activity, with kanamycin sulfate (KAN) exhibiting the strongest effect (2000 μM reduced luciferase expression to 22.15%, vs 74.62% for LNPs/siLuc).

**Figure 4:**
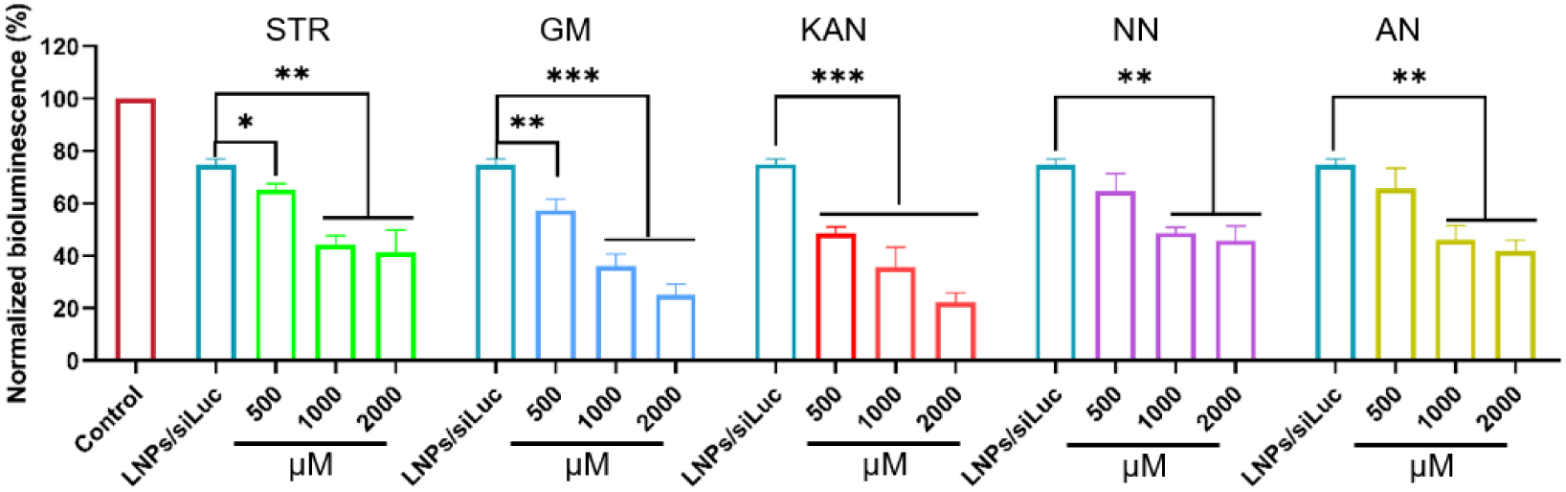
Inhibitory effect of different concentrations of aminoglycoside drugs on LNPs/siluc and Luc enzyme, The data presented as mean ± standard deviation (*n* = 3). *p < 0.05; **p < 0.01; ***p < 0.001.

### 3.5 Kanamycin Sulfate Enhances siRNA Lysosomal Escape

Given that aminoglycoside small-molecule drugs tend to accumulate in lysosomes and disrupt lysosomal function, we selected kanamycin sulfate (KAN) the most potent enhancer of siRNA delivery to investigate its effect on siRNA lysosomal escape. As shown in Figure 5A, in A549 cells at 4 h post-treatment, compared to LNPs/siRNA alone, the addition of KAN (1 mM) significantly reduced the colocalization of Cy5-siRNA with lysosomes (yellow puncta), while distinct free red fluorescent puncta (indicating escaped siRNA) were observed. In contrast, the LNPs/siRNA group still exhibited prominent yellow colocalization signals. By 8 h, the KAN-treated group showed even more pronounced free red fluorescence. Quantitative analysis of the colocalization coefficient (Figure 5B) confirmed a significant reduction in the LNPs/siRNA+KAN group versus LNPs/siRNA alone. In addition, I conducted co localization coefficients of lysosomes and siRNA at different concentrations of KAN. We found that when the KAN concentration was ≥0.5 mM, a clear reduction in siRNA–lysosome colocalization was observed (Figure S1), indicating enhanced lysosomal escape. These results demonstrate that KAN enhances endosomal escape of siRNA from LNPs, likely due to its disruptive effect on lysosomal membranes.

**Figure 5:**
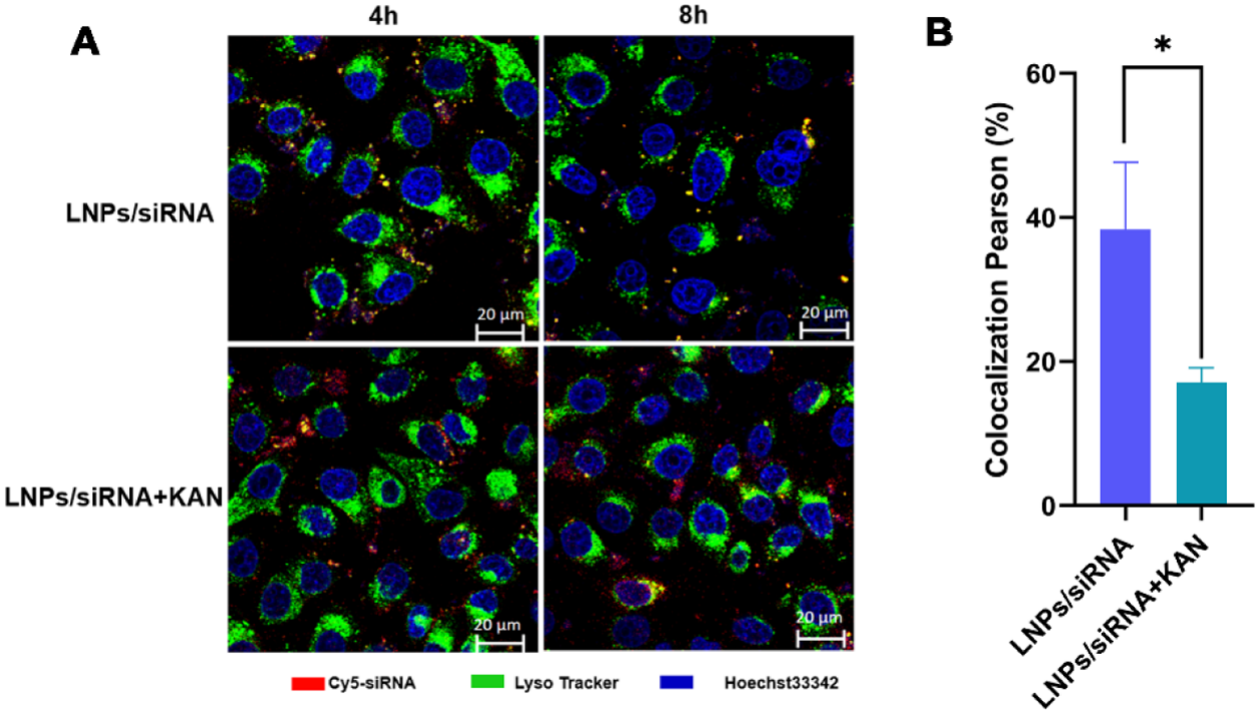
Lysosome escape effect of siRNA. (A) CLSM imaging of colocalization. Scale bars: 20 μm. (B) Quantitative analysis of colocalization. (siRNA: 50 nM, KAN: 1 mM). The data are presented as mean ± standard deviation (*n* = 3). *p < 0.05.

### 3.6 Kanamycin Sulfate Destabilizes Membrane Integrity

To elucidate the mechanism of KAN-facilitated endosomal escape, we evaluated its membrane destabilizing activity using a hemolysis assay with red blood cells (RBCs). As shown in Figure 6, at pH 7.4, neither LNPs/siRNA nor LNPs/siRNA+KAN induced hemoglobin release. However, under endosome mimetic acidic conditions (pH 6.2), the LNPs/siRNA+KAN group triggered significant hemoglobin release, with a 3.6-fold increase in hemolysis rate compared to LNPs/siRNA alone. This confirms that KAN selectively destabilizes membranes at acidic pH, promoting membrane rupture and cargo release.

**Figure 6:**
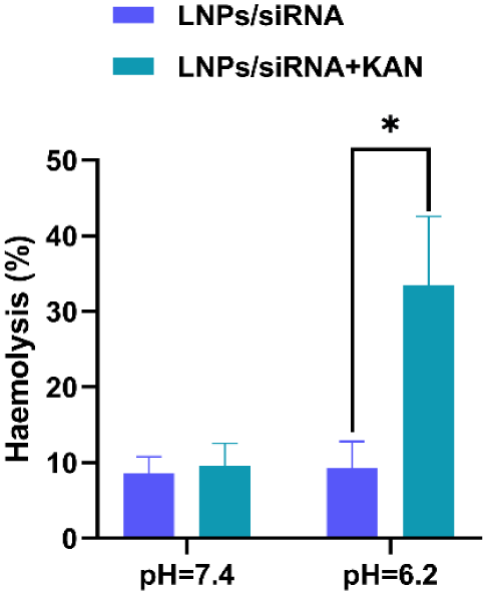
Hemolytic effect of nanoparticles at different pH values. The data presented as mean ± standard deviation (*n* = 3). *p < 0.05.

### 3.7 Kanamycin Sulfate Enhances EGFR mRNA Silencing

EGFR is overexpressed in tumors, driving proliferation and metastasis.^16^ To validate KAN’s delivery-enhancing effect, we used siEGFR-loaded LNPs to knock down EGFR mRNA. As shown in Figure 7A, LNPs/siEGFR+KAN markedly reduced EGFR mRNA levels compared to LNPs/siEGFR. This aligns with our earlier findings that KAN promotes endosomal escape, thereby improving siRNA bioavailability.

**Figure 7:**
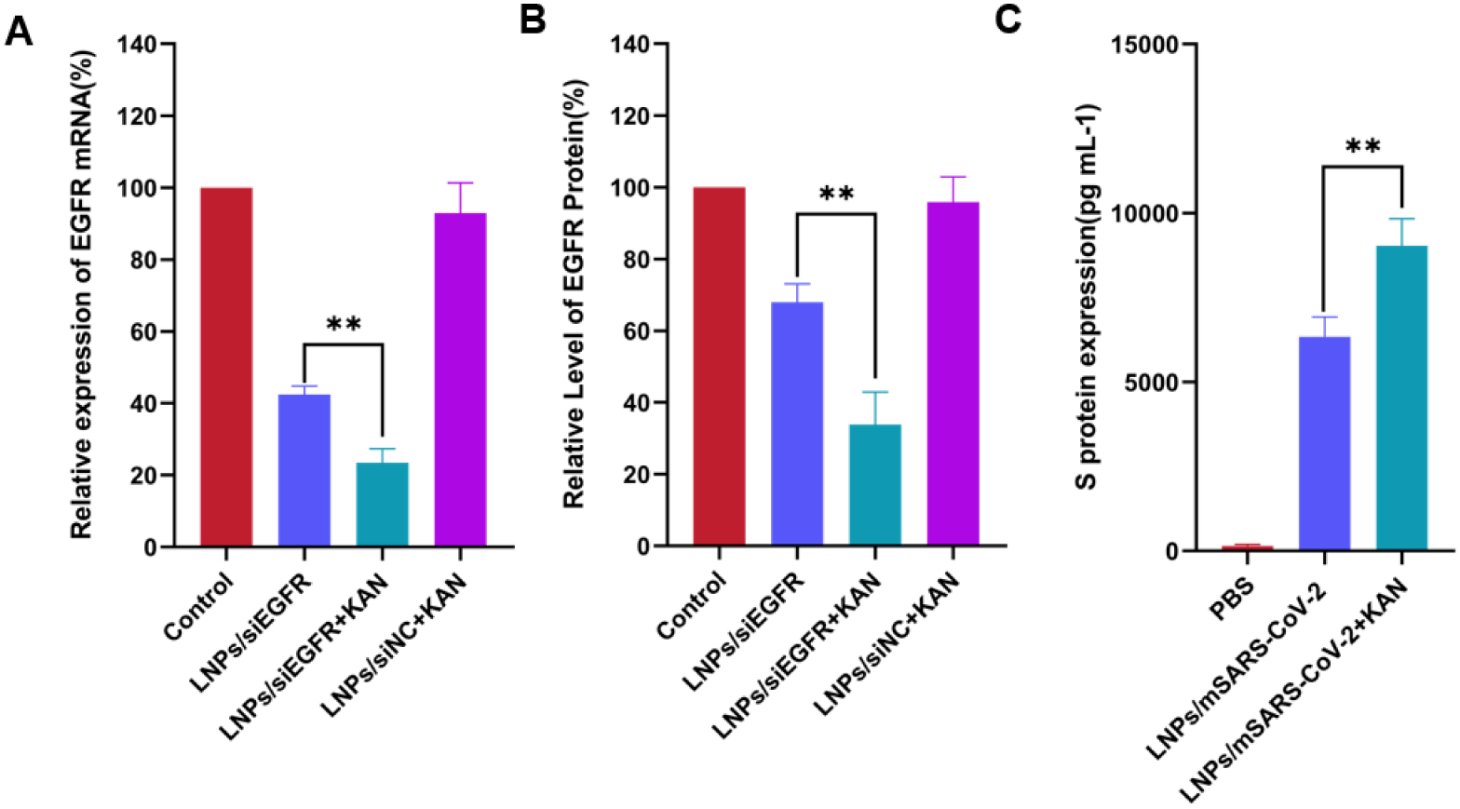
Kanamycin sulfate enhances the delivery effect of lipid nanoparticles at the gene and protein levels. (A) Relative expression levels of EGFR mRNA detected by PCR. (B, C) Relative expression levels of EGFR and S protein detected by ELISA Kit. The data are presented as mean ± standard deviation (*n* = 3). *p < 0.05; **p < 0.01.

### 3.8 Kanamycin Sulfate Potentiates EGFR Protein Expression

We further assessed KAN’s impact at the protein level using an EGFR ELISA. Consistent with mRNA data, LNPs/siEGFR+KAN significantly suppressed EGFR protein expression versus LNPs/siEGFR (Figure 7B).

### 3.9 Kanamycin Sulfate Boosts S Protein Expression via LNPs/mSARS-CoV-2

To evaluate KAN’s effect on mRNA delivery, we measured spike (S) protein expression in HEK293T cells transfected with LNPs/mSARS-CoV-2. ELISA revealed that LNPs/mSARS-CoV-2+KAN yielded higher secreted S protein levels in the supernatant compared to LNPs/mSARS-CoV-2 (Figure 7C).

## 4 Discussion

The clinical translation of nucleic acid drugs faces numerous challenges, with inefficient delivery being a core issue restricting their widespread application.^16–18^ Although lipid nanoparticles (LNPs), as the most mature delivery carriers currently available, have been successfully applied in siRNA drugs (e.g., Onpattro) and mRNA vaccines (e.g., COVID-19 vaccines), their lysosomal escape efficiency remains insufficient, leading to substantial degradation of nucleic acid drugs in lysosomes and failure to exert therapeutic effects.^19,20^ Therefore, developing novel synergistic strategies to enhance the delivery efficiency of LNPs has become a research hotspot in the field of gene therapy.

This study significantly improved the delivery efficiency of LNPs by introducing aminoglycoside small-molecule drugs as adjuvants. Experimental results showed that among various aminoglycoside drugs, kanamycin sulfate exhibited the strongest synergistic effect. Kanamycin (2000 μM) significantly enhanced the gene silencing efficiency of LNPs/siLuc, reducing Luc enzyme expression to 22.15%, compared to 74.62% in the group using LNPs/siLuc alone. Further mechanistic studies confirmed through red blood cell lysis assays that kanamycin significantly enhanced membrane-damaging effects under acidic conditions (pH 6.2), increasing hemoglobin release by 3.6-fold, while showing no such effect at physiological pH (7.4). This pH-dependent property highly matches the acidic microenvironment of endosomes/lysosomes, indicating that kanamycin can selectively act on endosomal membranes while avoiding nonspecific damage to the cytoplasmic membrane. Additionally, confocal microscopy observations revealed that the colocalization coefficient of Cy5-siRNA with lysosomes in the kanamycin-treated group was significantly reduced, further confirming its ability to promote lysosomal escape. The mechanism may involve the polyamine structure neutralizing the negative charge of the lysosomal membrane to reduce membrane stability. Frthermore, its hydrophobic moiety can insert into the membrane lipid bilayer, causing endosomal membrane permeabilization and facilitating siRNA escape. This finding is consistent with the reported mechanism of action of cationic amphiphilic drugs (CADs),^21^ but aminoglycosides offer greater advantages in biocompatibility and clinical availability.

In the siRNA delivery system, kanamycin, an outstanding member of aminoglycoside antibiotics, exerts its core mechanism in enhancing siRNA efficacy through efficiently promoting endosome/lysosome escape. This advantage stems from its unique structural features: the molecular net positive charge ranging from +3 to +4 forms a moderate cationic density, which not only enables effective binding with negatively charged siRNA and endosomal membrane phospholipids to initiate membrane perturbation, but also avoids excessive stability of complexes or cytotoxicity caused by overly high charges. The rigid “clamp-like” structure, formed by connecting two amino sugars with deoxystreptamine as the core, can precisely insert into the phospholipid bilayer, facilitating the generation of transient pores by inducing changes in membrane curvature and the formation of nonbilayer structures. Abundant hydroxyl groups enhance membrane interface interactions through hydrogen bond networks, further disrupting the stability of lipid arrangement. Meanwhile, its relatively hydrophilic property reduces nonspecific damage to the plasma membrane, concentrating its effect on the endosome/lysosome stage. This balance between structure and function allows it to achieve an optimal state in promoting escape efficiency, reducing toxicity, and ensuring siRNA release, thereby demonstrating the strongest siRNA efficacy enhancing effect among aminoglycosides.

This study not only demonstrated the synergistic effect of kanamycin on siRNA delivery but also extended it to the mRNA delivery field. In HEK293T cells, kanamycin significantly increased the expression level of the S protein of LNPs/mSARS-CoV-2, indicating that its synergistic effect is broad-spectrum and applicable to both gene silencing and protein expression nucleic acid drugs. This finding provides new insights for the development of mRNA vaccines and alternative therapies. Moreover, studies targeting the tumor therapy target EGFR showed that kanamycin significantly enhanced the silencing effect of LNPs/siEGFR on EGFR mRNA and protein, suggesting its potential application in cancer gene therapy.

Although aminoglycoside drugs show promising adjuvant potential, their long-term safety (e.g., ototoxicity, nephrotoxicity) still requires further evaluation [22]. Future research could focus on structural optimization (e.g., derivative design) to reduce toxicity or develop local delivery strategies (e.g., tumor targeting). Additionally, exploring other types of small-molecule adjuvants and their synergistic effects may provide more solutions for nucleic acid drug delivery.

This study provides important insights for the design of nucleic acid delivery systems. On one hand, novel lipid materials can be designed based on the structural characteristics of aminoglycosides to integrate membrane-damaging functions into LNPs; on the other hand, the combination of small-molecule adjuvants with other synergistic strategies (e.g., endosomal escape peptides or photothermal-triggered release) can be explored to achieve more precise delivery control. Furthermore, animal experiments and in vivo safety evaluations are the next research priorities to validate the translational potential of this strategy.

## 5 Conclusions

This study systematically elucidated the synergistic mechanism and application value of aminoglycoside small molecules (particularly kanamycin) as LNPs adjuvants. By disrupting endosomal membrane stability to promote lysosomal escape of nucleic acid drugs, this strategy significantly improves the delivery efficiency of siRNA and mRNA. This approach is not only simple to operate and cost-effective but also easily integrable with existing LNPs technologies, holding high clinical translation prospects. Through optimizing drug structures and delivery protocols in the future, aminoglycoside adjuvants are expected to become important tools in the field of nucleic acid drug delivery, driving the further development of gene therapy.

## Supplementary Material



## Data Availability

The data are available throughout the manuscript and supporting files.

## Notes

Figure S1. Colocalization Pearson correlation of siRNA with lysosomes at different KAN concentrations (PDF)
