# Supplementary material for: Aminoglycoside Drugs as Adjuvants to Enhance siRNA/mRNA Delivery by Lipid Nanoparticles
Source: ACS Omega. 2025 Nov 3;10(44):52442–8. doi: 10.1021/acsomega.5c05346 (PMC12612904; doi:10.1021/acsomega.5c05346)
Supplement: Supplementary file 1 [file ao5c05346_si_001.pdf]

Aminoglycoside Drugs as Adjuvants to Enhance siRNA/mRNA Delivery by Lipid Nanoparticles

*Xueru Sun *， Lei Qian*

Xueru Sun—Wulanchabu Medical College，Wulanchabu Inner Mongolia 012000，China

Lei Qian—Affiliated Hospital of Wulanchabu Medical College，Wulanchabu Inner Mongolia 012000,China

Corresponding author：

Xueru Sun

Wulanchabu Medical College，Wulanchabu，Inner Mongolia，China 012000，

Email：1149164689@qq.com

Author Contributions：Xueru Sun designed the study, supervised the study, and wrote and revised the manuscript. Lei Qian analyzed and statistically processed the data, and revised the manuscript.

Acknowledgements

This work was supported by Inner Mongolia Autonomous Region Education Science Research Fund (NZJGH2023113)

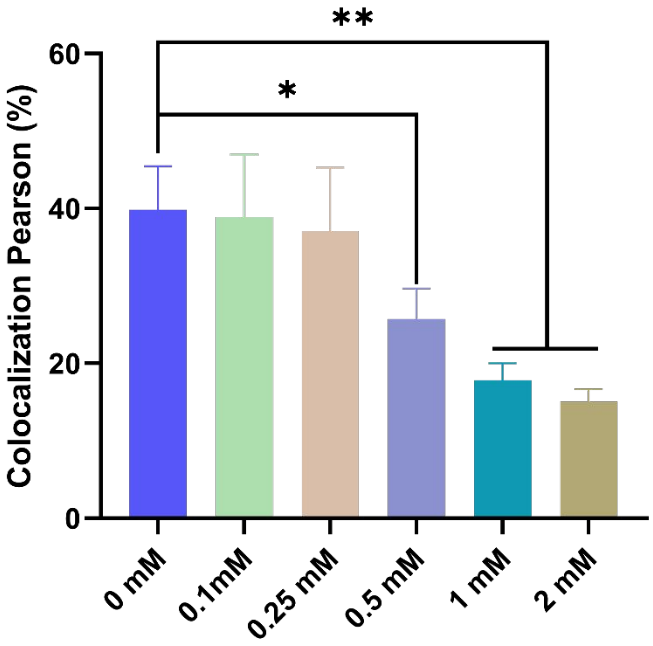

Figure S1. Colocalization Pearson of siRNA with lysosomes at different KAN concentrations. The data presented as mean ± standard deviation (n=3). *p < 0.05， **p < 0.01;
